# Validation of a Short Scale for Student Evaluation of Teaching Ratings in a Polytechnic Higher Education Institution

**DOI:** 10.3389/fpsyg.2021.635543

**Published:** 2021-07-05

**Authors:** Tarquino Sánchez, Jaime León, Raquel Gilar-Corbi, Juan-Luis Castejón

**Affiliations:** ^1^College of Electrical and Engineering, National Polytechnic School of Quito, Quito, Ecuador; ^2^Facultad de Ciencias de la Educación, Universdad de Las Palmas de Gran Canaria, Las Palmas de Gran Canaria, Spain; ^3^Department of Developmental Psychology and Didactics, University of Alicante, Alicante, Spain

**Keywords:** criterion validity, reliability, scale validation, short scale development, structure validity, student evaluation of teaching

## Abstract

The general purpose of this work is 2-fold, to validate scales and to present the methodological procedure to reduce these scales to validate a rating scale for the student evaluation of teaching in the context of a Polytechnic Higher Education Institution. We explored the relationship between the long and short versions of the scale; examine their invariance in relation to relevant variables such as gender. Data were obtained from a sample of 6,110 students enrolled in a polytechnic higher education institution, most of whom were male. Data analysis included descriptive analysis, intraclass correlation, exploratory structural equation modeling (ESEM), confirmatory factorial analysis, correlations between the short and long form corrected for the shared error variance, gender measurement invariance, reliability using congeneric correlated factors, and correlations with academic achievement for the class as unit with an analysis following a multisection design. Results showed four highly correlated factors that do not exclude a general factor, with an excellent fit to data; configural, metric, and scalar gender measurement invariance; high reliability for both the long and short scale and subscales; high short and long-form scale correlations; and moderate but significant correlations between the long and short versions of the scales with academic performance, with individual and aggregate data collected from classes or sections. To conclude, this work shows the possibility of developing student evaluation of teaching scales with a short form scale, which maintains the same high reliability and validity indexes as the longer scale.

## Introduction

The academic failure and dropout rates in higher education in Ecuador, especially in Engineering studies, are very high. Sandoval-Palis et al. (2020) find a dropout rate in the 1st year of university studies at the National Polytechnic School of around 70%. Braxton et al. (2000) and Kuh (2002) point out the quality of teaching as one of the determining aspects of academic failure and dropout. Likewise, instructional factors are one of the key factors in explaining academic success and dropout. Schneider and Preckel (2017) highlights the effect on academic readiness of the teacher-student interaction, the type of communication, the preparation, organization, and presentation of content by the teacher, the teacher's planning, and the feedback provided to the student, are some of the aspects.

Student evaluation of teaching (SET) ratings is a generalized procedure in the institutions of higher education (Richardson, 2005; Zabaleta, 2007; Huybers, 2014). SET is a useful tool for formative aims, such as feedback for the improvement of instruction, and for administrative decision-making about recruitment, career progress or economic incentives (Linse, 2017). A systematic review on the subject shows that there are very few publications on the validation of student evaluation of university teaching scales -SET- in South America, collected in the most important databases such as Scopus and WoS -Web of Science- (Pimienta, 2014; Andrade-Abarca et al., 2018), and some more when the scope of the search is expanded (Fernández and Coppola, 2008; Montoya et al., 2014).

In the Ecuadorian context, there are the works of Aguilar and Bautista (2015) and Andrade-Abarca et al. (2018), who validate questionnaires in the field of an Ecuadorian polytechnic university. While in the review by Loor et al. (2018) on the evaluation of university teaching staff, the need to improve the quality of the evaluation process is concluded.

### Student Evaluation of Teaching Ratings Scales

The instruments normally used to measure students' evaluation of their teachers, programs, and students' satisfaction with their instruction are known as standard rating scales. However, research on student evaluation of teaching ratings has not yet provided clear answers to some questions about their validity (Marsh, 2007a,b; Spooren et al., 2013; Hornstein, 2017; Uttl et al., 2017).

Many evaluation instruments have been constructed and validated within the home institution itself, and the results of such validation have not always been published, and in some instances they have not even been tested for psychometric quality (Richardson, 2005). In addition, there is a lack of consensus on the number and type of dimensions (Spooren et al., 2013), due to conceptual problems related to the lack a theoretical framework about what effective teaching is, and methodological problems concerning the measurement of these dimensions as a data-driven process (in which different *post-hoc* analytic techniques are used). It seems necessary to use the most common dimensions, which are associated with greater teaching effectiveness.

A question concerning construct validity that arises in relation to student evaluation of teaching rating scales is whether it has a one-dimensional (Abrami et al., 1997; Cheung, 2000) or multidimensional structure. Marsh et al. (2009) defended the application of exploratory structural equation modeling (ESEM) methods integrating confirmatory (CFA) and exploratory factor analyses (EFA) to analyse issues related to multidimensional student evaluations of university teaching (SETs), on the basis of the measures that can be obtained both of the specific dimensions and a general factor of the quality of teaching.

An open and controversial question related to the criterion validity is the relationship of SET scores to student academic achievement. To answer this question, a series of revision and meta-analytical studies have been carried out (Cohen, 1981; Feldman, 1989; Clayson, 2009; Uttl et al., 2017). Taken together, the results regarding the relation between SET and academic performance, when multiple sections are included and the previous academic achievement is controlled, show that SET is moderately related to academic achievement; however, the effect of SET on academic performance is smaller than that found in some previous meta-analytic studies (Cohen, 1981; Feldman, 1989), at around only 10%.

Another methodological question concerns evaluation systematic-bias. This problem is present when a confirmed characteristic of students habitually influences their evaluations of teachers (e.g., gender; Centra and Gaubatz, 2000; Badri et al., 2006; Basow et al., 2006; Darby, 2006; Boring, 2015). A possible source of bias is the discipline. If the evaluation of teaching is situational and is affected by academic disciplines, being higher in studies in the field of education and the liberal arts and less in other areas such as business and engineering (Clayson, 2009), it seems necessary to carry out new studies in areas different from the previous ones, such as the technical areas where there are fewer studies on the subject.

The present study was carried out in a different context to most previous studies (Clayson, 2009), the student evaluations of teaching in a higher education institution, the National Polytechnic School of a South American country, Ecuador, where students study technical subjects, such as engineering, architecture, and biotechnology. Unfortunately, in South America there is a shortage of reliable and valid SET scales in polutechnic higher education institution, although it is a widespread procedure in these institutions since the early 1980's (Pareja, 1986).

The Council of Ecuadorian Higher Education establishes the obligatory nature of the evaluation of the teaching staff of higher education institutions, both for their entry and for their promotion, in the Career and Ladder Regulations of the Professor and Researcher of the Higher Education System, and they may even be dismissed from teaching in case of performance evaluations of <60% twice consecutively, or four comprehensive evaluations of performance <60% during their career (Consejo de Educación Superior, 2017).

The evaluation of the quality of teaching in the National Polytechnic School of Ecuador uses different procedures, including self-assessment, evaluation by peers and managers, and evaluation by students through evaluation questionnaires. The elaboration of this questionnaire is based on the criteria proposed by the institution itself and the guidelines suggested by the Higher Education Council (Consejo de Educación Superior, 2017).

The instrument of student evaluation of teaching used in the National Polytechnic School is the “Cuestionario de Evaluación de la Enseñanza del Profesor de la Escuela Politécnica Nacional del Ecuador” (Teacher Evaluation Questionnaire of the National Polytechnic School). The elaboration of the questionnaire was based on previous SET literature (Toland and De Ayala, 2005; Marsh, 2007a; Mortelmans and Spooren, 2009) and consists in the proposal of several effective teaching criteria. Next, a teaching committee, part of the management team of the National Polytechnic School, developed a set of items. This committee consisted of 5 main tenured professors with extensive experience in teaching quality, and a representative from the administrative sector and a student. The aspects to be evaluated and the specific items that make up the questionnaire are approved each academic year by the management team of the National Polytechnic School. The items are grouped theoretically into the following four factors. 1. Planning, mastery, and clarity in the explanation of the subject matter (i.e., The teacher conveniently expresses the class objectives and contents, indicating their relationship with the student's training). 2. Methodology and resources (i.e., The teacher prepared teaching material apart from the textbook and made it known). 3. Teacher-student relationship (i.e., The teacher created a climate of trust and productivity in class). 4. Evaluation (i.e., The evaluation events are related to the teaching given). Although the number and dimensions of effective teaching remains an open question (Spooren et al., 2013), these four dimensions are present in the most of SET literature (Feldman, 1989; Richardson, 2005; Huybers, 2014).

Thus, face and content validity are taken into account during the process of developing an instrument. Face validity indicates whether an instrument seems appropriate, that is, face validity does not analyze what the instrument measures but what it appears to measure; i.e., the extent to which the items of a SET instrument appear relevant to a respondent (Spooren et al., 2013; Rispin et al., 2019). Content validity refers to whether the content of an instrument has been included in an exhaustive and representative way, that is, if the content has been included in an appropriate way. Content validity is obtained from the consensus based on informed opinion of experts; it is recommended to include at least five experts for the evaluation of content validity (Yaghmale, 2009). However, the empirical validation is minimal and is limited to a descriptive analysis of the items individually considered. It lacks a complete process of construct and criterion validity, as well as an estimation of the reliability of the scale and/or the subscales that make up these questionnaires.

Although many studies have been developed on the subject of the validation of student evaluation of teaching scales in higher education, few have done so in the specific scope of polytechnic institutions and SEM studies; there are also very few examples of rigorous development of short teacher assessment scales. For this reason, our work tries to contribute to filling this gap.

### Scale Reduction

Currently, a line of work has been developed to reduce the length of scales already used or elaborate scales with a reduced number of items. The lack of time for the application of scales, fatigue, and possible stereotyped responses in scales that are too long or that are part of a set of scales that are applied within the same study, etc., has led to proposals of short scales (Gogol et al., 2014; Lafontaine et al., 2016). These scales have to be small enough to allow for a rapid assessment of purposed constructs, but large enough to ensure appropriate reliability, validity, and accurate parameter estimation.

Short scales are considered to present psychometric inconveniences in comparison to long scales with regard to both reliability and validity, as they can be more affected by random measurement errors (Lord and Novick, 1968; Credé et al., 2012).

In the short-form scales, the number of items per factor proposed varies from one to four items. Thus, several authors propose scales and subscales in which each factor should include four items (Marsh et al., 1998, 2009, 2010; Poitras et al., 2012). Moreover, other authors, such as Credé et al. (2012), point out the loss of psychometric qualities when the scales have between one and three items. On the other hand, Kline (2016) points out that construct validation procedures, such as confirmatory factor analysis and other modeling methods, require at least three indicators per factor for a model to be identified. From a point of view that combines theoretical demands with practical interest, the PISA study of 2000 and the German PISA study of 2003 use short scales with three items (Brunner et al., 2010).

Another group of studies propose the use of short scales based on the finding that reliability and validity of short measures is similar to those of the corresponding longer scales measures, and have high correlation with long scales (Nagy, 2002; Christophersen and Konradt, 2011; Gogol et al., 2014). Gogol et al. (2014) compared the reliability and validity of three-item and single-item measures to those of the corresponding longer scales, finding satisfactory reliability and validity indices in all short forms and a high correlation with long scales; however, single-item measures showed the lowest reliability indices and correlations with the longer scales. Based on these results, the authors defended the use of short scales.

In sum, there are empirically founded reasons to propose short scales of three or four items. Although three items seem sufficient to guarantee the reliability and validity of the measure, in some cases, such as when additional assumptions are made about the psychometric properties of the items and factors (variables error variances, factor variances, etc.) or the hierarchical nature of the data is taken into account in multilevel analysis, four items per factor are recommended for accurate parameter estimation (Marsh et al., 1998).

### Research Objectives

Hence, in this work, the following objectives are established:

1. Validate a Student Evaluation of Teaching Rating Scale and a short version of the corresponding long scale, including four items for each measured dimension, in a large sample of higher education students enrolled in a polytechnic higher education institution.
2. Test alternative structures of the dimensions of the Student Evaluation of Teaching Rating Scale.
3. Find the relationship between the long and short forms of the scale and academic achievement.
4. Examine whether the scores are invariant with respect to relevant variables such as the gender of the students in the context of scientific-technological studies.
5. Considering the hierarchical nature of the data, determine the ratings of the teaching of individual students located in different groups, classes, or sections, as well as where each group evaluates a different teacher.

## Materials and Methods

### Participants

The sample comprised 6,110 students of the National Polytechnic School of Ecuador who rated the teaching of their teachers. These students were enrolled in eight different faculties in 28 different degree programs and attended 358 different classes. 68.3% of the students were male and 31.7% female. The higher percentage of male students is representative of the population of students of polytechnic studies. The average age was 22.6 years old (SD = 3.2). These students rated the teaching of their teachers during the 2016–17 academic year.

The sample of teachers was composed of 310 teachers, most of which were males (62.8%), aged between 26 and 57 years (mean = 43.7), belonging to all professional categories, from assistant professor to principal, with a majority (42%) of full professors, and extensive teaching experience (mean = 18,6 years).

This sample of participants corresponds to the students enrolled in the aforementioned studies, who took part in the evaluation process of the teaching staff of their institution, the EPN, at the end of a semester.

### Measures

Students' evaluations of teaching ratings were obtained from the “Cuestionario de Evaluación de la Enseñanza del Profesor de la Escuela Politécnica Nacional del Ecuador” [Teacher Evaluation Questionnaire of the National Polytechnic School], approved by the teaching staff for the 2016–17 academic year. This scale comprises 32 items grouped theoretically into the following four factors. 1. Planning, mastery, and clarity in the explanation of the subject matter (items 1–9). 2. Methodology and resources (items 10–15). 3. Evaluation (items 16–23) 4. Teacher–student relationship (items 24–32). Response scale ranges from 1 to 5; 1: do not agree at all; 2: little agreement, 3: moderately agree; 4: strongly agree; and 5: totally agree. The full and reduced scales with the items grouped into the four theoretical dimensions are included in the Appendix A.

The measures of student academic performance were obtained for a subsample of 1538 students. This subsample consisted of those students for whom data on their academic performance were available in the university's administrative computerized records. There is no known evidence that this subsample is biased with respect to the total sample used in this study. This measure of academic performance at the end of the semester was operationalized by the grade awarded by the teacher, based on a final exam: a written examination, both theoretical and practical. These final exams were the same across sections in some cases and were different for different sections in others. Different sections follow the same program and have the same assessment criteria that are specified in the study program of each course. Therefore, the exams, although different, can be considered quite equivalent. There are also common general rules for all exams in the National Polytechnic School of Ecuador. The scores of final grades ranged from 0 to 40 for all courses.

Students' age and gender, as well as teachers' age, gender, and experience, were collected from administrative records.

### Procedure

The data were collected from the existing computer records in the administration of the Polytechnic School, and permission for access to them was granted to the academic staff of the Institution. The data provided by the institution were anonymous, with only one identification code for each student.

The application of the evaluation of teaching scale by the students was carried out toward the end of the semester, before they knew their final grades. All the teachers were evaluated by the students in a similar period of time. All the students had to evaluate the teachers to be able to access their final grades. The student evaluation of teaching was conducted through an electronic platform on which the data were recorded.

The impact that faculty procedures of student evaluations of teaching have on response rates has been analyzed by several authors in special electronic evaluations. Thus, Young et al. (2019) found that evaluations made by students were considerably higher when faculty gave in-class time to students to complete student evaluation of teaching, compared to an electronic form issued by the administration. However, other studies of this issue did not find differences between the evaluations made with electronic questionnaires and paper and pencil questionnaires, or when a more representative sample responded instead of a smaller, more biased sample (Nowell et al., 2014).

As response rates to electronic administration are lower than to paper-and-pencil questionnaires, the procedure followed in this work consisted in requiring all the students to answer the evaluation survey in order to access their final grades. This procedure has proved useful and valid in some higher education institutions (Leung and Kember, 2005; Nair and Adams, 2009).

### Data Analysis

#### Preliminary Analyses

We explored means, standard deviations, skewness, and intraclass correlations (ICCs) for all items. Skewness indicates the asymmetry of the distribution, while ICC gives information about the non-independence of data, that is, the similarity of students' responses in the same class.

#### Construct Validity

To gather evidence of the scale's construct validity, we followed the recommendations of Schmitt et al. (2018). There are different methods to retain the “best” factor structure; for instance, exploratory factor analysis (EFA), confirmatory factor analysis (CFA), or exploratory structural equation model (ESEM). EFA has the disadvantage of the difficulty to replicate results with different samples, while CFA leads to biased loadings and correlations between factors because it requires that cross-loadings be 0 in the non-target factors (Garn et al., 2018). ESEM combines EFA and CFA, provides goodness of fit indices, and allows testing for multiple-group measurement invariance (Xiao et al., 2019). Schmitt et al. (2018) recommend using EFA when there is no a priori theory, using CFA when there is a strong theory and evidence of the scale structure, and using ESEM when the a priori theory is sparse. Howard et al. (2018) add that ESEM should be retained over CFA when correlations are different between factors are different in these two methods.

Another interesting issue in factor analysis, specifically in multidimensional structures, is bi-factor models (Morin et al., 2016). Bi-factor models are used to divide covariance between a global factor (i.e., teachers' style) and specific factors (i.e., Methodology and resources or Teacher-student relationship).

**Figure 1:**
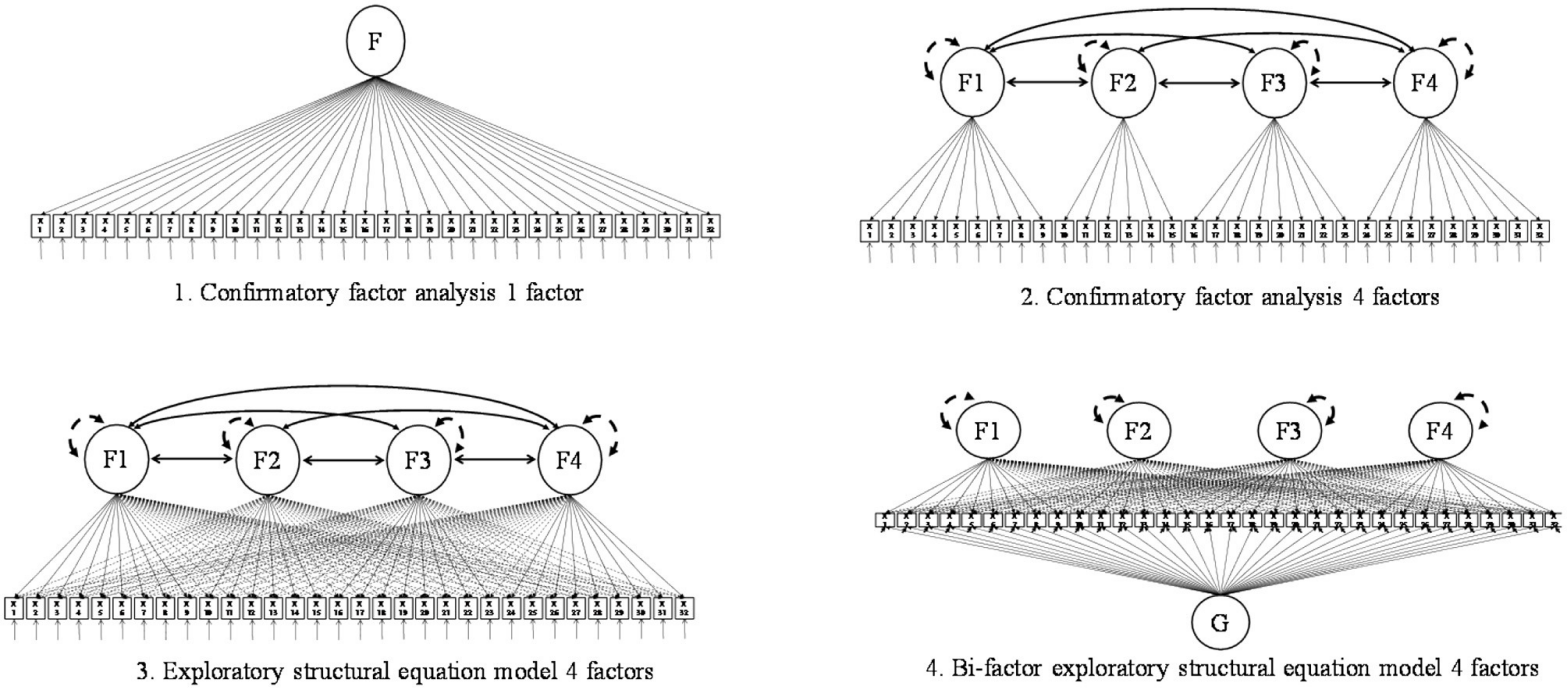
Proposed structural factors of the model tested.

Therefore, in view of the above information and our data, we can test the following models: one-factor *via* CFA, four-factor *via* CFA, four-factor *via* ESEM, and four- and bi-factor *via* ESEM (see Figure 1). To select the factor structure, we relied on the adjusted χ^2^-difference tests and changes in CFI and RMSEA. The estimation method was Robust Maximum Likelihood because the data were non-normal; moreover, as responses were not independent, we corrected χ^2^ and standard errors using a sandwich estimator (Muthen and Satorra, 1995; Muthén and Muthén, 2020). All analyses were conducted with Mplus 8.4 (Muthén and Muthén, 2020).

#### Short Version

To choose items for a short version, we account for factor loadings, corrected for item-test correlations, reliability, and the item theoretical significance (Marsh et al., 2010). To test the agreement of both versions, we relied on the Levy correction of the short vs. long form correlation. This correction accounts for the shared error variance between both forms due to the subset of items (Levy, 1967; Barrett, 2015). Moreover, because correlation only considers the monotonicity between both forms, we also relied on the Gower index (Gower, 1971; Barrett, 2012), whose values range between 0 and 1, where values close to 1 indicate agreement.

#### Gender Measurement Invariance

To test whether male and female students interpret the scale similarly, we performed a measurement invariance test (Vandenberg and Lance, 2000). Specifically, we compared three models: configural, metric, and scalar (Muthén and Muthén, 2020). The configural model has factor loadings, intercepts, and residual variances free across groups and factor means fixed at zero in all groups. In the metric model, factor loadings are held equal across groups, while intercepts and residual variances are free across groups, and factor means are fixed at zero in all groups. Finally, in the scalar model, factor loadings and intercepts are equal across groups, while residual variances are free across groups, and factor means are constrained to zero in one group and free in the other group. For model comparisons, we used the adjusted χ^2^-difference tests and changes in CFI and RMSEA.

#### Reliability

Finally, to test the reliability of the short and long form, we did not use Cronbach's alpha because there is increasing evidence of its lack of accuracy and the difficulty of meeting its assumptions: the parallelism and tau-equivalence of the items (Zhang and Yuan, 2016; McNeish, 2018). Cho (2016) proposes different formulas to estimate reliability whenever items lack parallelism, tau-equivalence, or both, not only for unidimensional structures but also for multidimensional structures.

#### Criterion Validity: Relation With Academic Achievement

To analyse the relationships between student ratings of teaching and academic performance, the data were taken individually and grouped into sections. Initially, the validity of students' ratings might be evidenced by the correlation between SET and academic achievement. Nevertheless, students' grades cannot be supposed to constitute a simple measure of teaching effectiveness because each group could have different evaluations (Richardson, 2005). The key evidence cited in support of student evaluations of teaching as a measure of a teacher's instructional effectiveness is multisection studies, in which different professors teach the same subject following the same outline, and at the end of the semester, all the sections have the same exam or equivalent ones (Cohen, 1981; Uttl et al., 2017). To find the correlation between scale scores and academic performance, the data were taken individually and treated as a typical multisection study in which the average class was used as the unit of analysis.

## Results

### Preliminary Analyses

Means varied between 3.85 for Item 15 and 4.07 for Item 9, while standard deviations ranged from 1.02 for Item 2 to 1.16 for Item 11. Skewness varied from −0.840 for Item 15 to −1.120 for Item 1. More information can be found in Appendix B.

### Construct Validity

We compared the four proposed models. We observed that the probability that a four-factor CFA had the same fit as a one-factor CFA was *p* < 0.001 (Δχ^2^ = 10217.93, df = 8). Similarly, the probability that a four-factor ESEM had the same fit as a four-factor CFA was *p* < 0.001 (Δχ^2^ = 1272.977, df = 84). Finally, the probability that a four-factor ESEM had the same fit as a bi-factor four-factor ESEM was *p* < 0.001 (Δχ^2^ = 1143.317, df = 28).

The structure with the best fit was the bi-factor four-factor ESEM (see Table 1). However, to retain this structure, moderate-high factor loadings were required in the global factor (Howard et al., 2018), and in this case, the factor loading absolute values were between 0.024 and 0.228, with an average value of 0.093. Therefore, we discarded the bi-factor four-factor ESEM and proceeded to explore the four-factor ESEM structure. This structure provided moderate to high loadings and low cross-loadings (see Appendix A). Specifically, for Planning, mastery, and clarity in the explanation of the subject matter (Factor 1), the loadings ranged between 0.508 and 0.857, for Methodology and resources (Factor 2) between 0.601 and 0.856, for Evaluation (Factor 3) between 0.385 and 0.885, and for Teacher-student relationship (Factor 4) between 0.629 and 0.958. Thus, we decided to retain this structure.

**Table 1:** χ^2^-test and fit indices for different structures.

**Model**	*****χ*****^**2**^		**RMSEA**	**CFI**
	**Value**	**DF**		
CFA 1F	16679.456	464	0.076	0.873
CFA 4F	6461.526	458	0.046	0.953
ESEM 4F	5188.549	374	0.046	0.962
Bi-ESEM 4F	4045.232	346	0.042	0.970

### Short Version

#### Construct Validity

Following Marsh et al.' recommendations (2010), we selected four items of each subscale. Next, we proceeded to test the selected structure *via* ESEM. The chi square test result and fit indices were: χ^2^(6110, 62) = 509.115 (*p* < 0.001), CFI = 0.992, RMSEA = 0.034 (90% C. I. = 0.032, 0.037). For Planning, mastery, and clarity in the explanation of the subject matter, the loadings ranged between 0.676 and 0.898, for Methodology and resources between 0.572 and 0.916, for Evaluation between 0.672 and 0.864, and for Teacher-student relationship between 0.675 and 0.946 (see Appendix C).

#### Agreement Between Both Versions

As shown in Table 2, Levy's corrected correlation and the Gower index revealed a high concurrence between both forms, ranging from *r* = 0.893 to *r* = 0.974.

**Table 2:** Agreement between the long and short forms.

**Factor**	**Levy's correlation**	**Gower index**
Planning, mastery, and clarity	0.893	0.963
Methodology and resources	0.901	0.974
Evaluation	0.919	0.972
Teacher-student relationship	0.918	0.969

### Gender Measurement Invariance

#### 32-Item Scale

Multiple-group analyses to examine potential gender differences in the model results showed that the probability of the same fit between the configural and the metric model was *p* < 0.902 (Δχ^2^ = 93.127, df = 112). Similarly, the comparison between the metric and the scalar model yielded *p* < 0.902 (Δχ^2^ = 126.335, df = 140). Thus, we found no gender differences in loadings, thresholds, or factor means in the long form scale (see Table 3).

**Table 3:** χ^2^-test and fit indices for invariance testing.

**Model**	*****χ*****^**2**^		**RMSEA**	**CFI**
	**Value**	**DF**		
Configural	2301	748	0.051	0.959
Metric	2321	860	0.046	0.961
Scalar	2374	888	0.046	0.960

#### 16-Item Scale

The comparison between the configural and the metric models revealed that the probability that the model fits would be the same was *p* < 0.847 (Δχ^2^ = 38.043, df = 48). Similarly, the comparison between the metric and the scalar model yielded *p* < 0.629 (Δχ^2^ = 55.838, df = 60). Thus, we did not find gender differences in loadings, thresholds, or factor means in the short form either (see Table 4).

**Table 4:** χ^2^-test and fit indices for invariance testing (short form).

**Model**	*****χ*****^**2**^		**RMSEA**	**CFI**
	**Value**	**DF**		
Configural	274.8	124	0.039	0.980
Metric	305.6	172	0.031	0.991
Scalar	327.8	184	0.032	0.990

### Reliability

#### 32-Item Scale

The reliability of the scale was assessed using the Congeneric Correlated Factors formula. Reliability for the whole scale was 0.980, for Planning, mastery, and clarity in the explanation of the subject matter 0.949, for Methodology and resources 0.901, for Evaluation 0.948, and for Teacher-student relationship 0.947.

#### 16-Item Scale

The reliability for the whole scale was 0.972, for Planning, mastery, and clarity in the explanation of the subject matter 0.904, for Methodology and resources 0.901, for Evaluation 0.920, and for Teacher-student relationship 0.919.

### Correlation With Academic Achievement

Table 5 shows the correlations between the long and short versions of the scale of evaluation of teaching with academic performance, taking individual and aggregate data in sections. As we can see, all the correlations were statistically significant with moderate-low values. Both the subscales and the total scale showed significant correlations with academic performance. The values of the correlations of the reduced scale were very similar to those of the long scale. In addition, the correlations in the aggregated data in classes or sections were slightly higher than in the individual data.

**Table 5:** Correlations between the long and short versions of the scale of evaluation of teaching with academic performance, taking individual, and aggregate data in sections.

**Subscales**	**Individual data**		**Aggregate data in sections**	
	**Long**	**Short**	**Long**	**Short**
1. Planning, explanation, and presentation of subject	0.21	0.21	0.21	0.23
2. Method and materials	0.23	0.22	0.26	0.26
3. Evaluation	0.23	0.22	0.24	0.23
4. Teacher-student relationship	0.21	0.20	0.26	0.23
Total scale	0.23	0.23	0.25	0.26

*All correlations showed a p < 0.01*.

## Discussion

The results clearly show the structural validity of the student evaluation of teaching ratings elaborated in the National Polytechnic School of Ecuador. Given that the main objective of this study is to propose a short scale that shows reliability and validity, AFC and Exploratory Structural Equation Modeling were used.

Results showed a multidimensional model with four highly correlated factors that do not exclude a general factor, with an excellent fit to data, both in the long scale and in the short version of the scale. The structure with the best fit was the bi-factor four-factor ESEM; however, the factor loadings on the global factor were low (Howard et al., 2018) and, thus, the four-factor ESEM structure was retained.

Based on a sample of 26,746 students who took the Program for International Student Assessment (PISA) of 2012, Scherer et al. (2016), found that bi-factor exploratory structural equation modeling outperformed alternative approaches with respect to model fit.

The researchers are divided on the basis of the existence of a second-order general factor (Abrami et al., 1997; Cheung, 2000) or different first-order correlated factors (Marsh, 1991b, 2007a). As for the practical implications of this issue, perhaps the most accurate conclusion is the one provided as early as 1991 by Marsh (1991a) himself: “I have chosen a middle ground recommending the use of both specific dimensions and global ratings” (p. 419).

The use of academic performance measures as an external criterion validity of the student evaluation of teaching (SET) rating scales is very common in validation works, which has been called a strong test for criterion validity. However, the meta-analyses (Cohen, 1981; Feldman, 1989; Marsh, 2007a; Clayson, 2009; Uttl et al., 2017) shows the existence of moderate (0.50–0.20) to small (0.20–0.00) positive correlations between SET scores and student achievement. Although these results provide relative evidence of the convergent validity of SET scales; due to the variety of views concerning good teaching, and due to the variety in the measurement and predictors of student achievement (Spooren et al., 2013; Schneider and Preckel, 2017), academic achievement should not be the only indicator of SET scales criterion validity.

Student Evaluation of Teaching rating scales are multidimensional, many researchers defend the use of single, global scores (Apodaca and Grad, 2005). For this reason, even when recognizing the multidimensional and hierarchical structure of the dimensions evaluated in the scales on student evaluation of teaching, many works studying this issue use global scores; meanwhile, the feedback provided to teachers for the improvement of teaching practice includes a profile of the scores in the different dimensions, which show the strengths and weaknesses of each teacher's methods.

Given the existence of student gender bias in student evaluation of teaching, configural, metric, and scalar gender measurement invariance were tested. Previous research has shown that female subjects are likely to score higher in SET ratings (e.g., Badri et al., 2006; Darby, 2006). Bonitz (2011) found that gender variations in SET scores could be due to gender variations in traits such as agreeableness that correlate with the SET scores. However, the results of this study showed configural, metric, and scalar gender measurement invariance in the context of scientific-technological studies.

Although the literature on gender bias in SET shows that male students express a bias in favor of male professors (Centra and Gaubatz, 2000; Boring, 2017; Mitchell and Martin, 2018; American Sociological Association, 2019), the extensive review by Kreitzer and Sweet-Cushman (2021), shows that the effect of gender is conditional upon other factors. Other works show that the gender bias against perceived female instructors disappears (Uttl and Violo, 2021). The results of Rivera and Tilcsik (2019) even show that these gender differences can disappear in scales with six points or less, like those of our scale.

The results of this work also show the concurrent validity of the reduced scale of 16 items, which showed a high correlation with the full scale of 32 items. Levy's corrected correlation and the Gower index revealed high concurrence between both forms, with values above 0.90. These results are slightly higher than those obtained in other studies that also showed a high degree of agreement between long and short forms of such scales (Gogol et al., 2014; Lafontaine et al., 2016).

The high values of the reliability coefficients, estimated according to the assumptions of the SEM model used, are also striking for both the long and short whole scales and subscales. These values were higher than 0.90 and reached values of 0.98 and 0.97 for the whole scales. The Congeneric Correlated Factors procedure (Cho, 2016) was applied in consideration of there being different factor loadings to obtain the values of multidimensional reliability coefficients apart from Cronbach's alpha, which supposes that all factor loadings are equal (i.e., tau-equivalents), and thus underestimates the reliability.

On the other hand, the results also showed moderate, significant correlations between both the long and short versions of the scale with academic performance, taking individual and aggregate data in classes or sections.

The evidence in support of student evaluations of teaching as a measure of teachers' instruction effectiveness comes from studies showing correlations between measures of student evaluation and student achievement, a strong test for criterion validity.

The results obtained with aggregate data, taking the section as the unit of analysis, showed a moderate and statistically significant correlation (0.26) between student ratings and final performance. This result is expected from studies of instructors' teaching effectiveness, in which it is considered that multisection studies are more appropriate for apprehending the true relationship between student evaluations of teaching and academic performance (Cohen, 1981; Uttl et al., 2017).

However, the relationship of the students' evaluation of teaching with their academic performance is lower than that found in some previous meta-analytic studies (Cohen, 1981), but higher than that found in the meta-analysis of Uttl et al. (2017) of the studies published to that date, when small study size effects and prior academic achievement were considered. Taken together, the results demonstrated the good psychometric qualities of the Teacher Evaluation Questionnaire of the National Polytechnic School and its construct and criterion validity, as well as its high reliability. In addition, the psychometric indices of the short version of this scale suggest the possibility of developing short scales of three or four items that are equally reliable and valid.

In addition, the relationships obtained between the long and short versions of the new instrument with academic performance have practical implications for teacher teaching. This instrument may help teachers to adapt their teaching to student needs and preferences in the context of specific characteristics of polytechnic studies.

However, we must not lose sight of the open controversy between students' perceptions of the quality of the teaching, or perceptions of leaning, and their actual learning. In the context of STEM -Science, Technology, Engineering and Mathematics—instruction Deslauriers et al. (2019) find that students in active classrooms learned more, but their perception of learning was lower than that of their peers in passive instruction.

Regarding the limitations of this study and possible future studies, given that the long and short forms were administered as part of the full scale, and despite the correction of Levy and Gower for the calculation of the correlation between the two version, it would be necessary to administer the long and short scales to the same sample independently. In addition, it would be convenient to examine the factorial structure of the short scale in an independent representative sample of students. In this study, we analyzed the relationship with academic achievement, it might be of interested to explore the relationship with higher education engagement (Vizoso et al., 2018) or general pedagogical knowledge (Klemenz et al., 2019). Finally, obtaining longitudinal data in the same and different samples of the National Polytechnic School could serve to deepen the validity of the scale developed in this work.

It should also be taken into account that these results have been obtained in a single institution, which limits the generality of the results; however, it is the largest institution of polytechnic studies (science, biotechnology, engineering, architecture, etc.), the largest in Ecuador that collects students from all over the country.

In sum, this work provides evidence of the validity of a teaching evaluation scale in the setting of a polytechnic institution of higher education, as well as a rigorous methodological procedure for the validation of short versions of teaching evaluation scales.

## Data Availability Statement

The raw data supporting the conclusions of this article will be made available by the authors, without undue reservation.

## Author Contributions

TS: project administration and data curation. JL: methodology and writing review and editing. RG-C: conceptualization and resources. J-LC: supervision and writing original draft. All authors contributed to the article and approved the submitted version.

## Conflict of Interest

The authors declare that the research was conducted in the absence of any commercial or financial relationships that could be construed as a potential conflict of interest.
