# Supplementary material for: Validation of a Short Scale for Student Evaluation of Teaching Ratings in a Polytechnic Higher Education Institution
Source: Front Psychol. 2021 Jul 5;12:635543. doi: 10.3389/fpsyg.2021.635543 (PMC8287253; doi:10.3389/fpsyg.2021.635543)
Supplement: Supplementary file 1 [file Data_Sheet_1.DOCX]

Annex (English)

**Planning, mastery, and clarity in the explanation of the subject matter**

1. The teacher conveniently expresses the class objectives and contents, indicating their relationship with the student’s training *

2. The teacher appropriately selects the class activities according to the objectives *

3. The teacher is clear in his explanations and presentations

4. The teacher links fundamental theoretical concepts and principles with practice *

5. The teacher solves the difficulties that arise

6. The teacher shows mastery of the subject *

7. The teacher demonstrates in the classroom that he plans his classes in advance

8. The teacher is creative and dynamic in class

9. The teacher shows that he is up to date on the subject that he teaches

**Methodology and resources**

10. The teacher prepared teaching material apart from the textbook and made it known

11. The teacher organizes didactic experiences such as visits, excursions, projects, and discussions *

12. The complementary material recommended or used by the teacher is interesting *

13. The teacher uses methods that favour learning *

14. The teacher conveniently uses different teaching methods

15. The teacher uses a varied methodology *

**Evaluation**

16. The teacher has explained the methods of course evaluation

17. The teacher has used objective methods to evaluate the students *

18. The teacher has used evaluations to reorient student learning

19. The teacher has taken into account aspects that are not merely cognitive in the evaluation

20. The teacher has evaluated students fairly and impartially *

21. The teacher has explained the minimum level required to pass the course, and why *

22. The objectives that were intended to be achieved have been defined in a clear and concise manner

23. The evaluation events are related to the teaching given *

**Teacher-student relationship**

24. The teacher checked that the students understood what they were taught

25. The teacher encouraged initiative on the part of the students

26. The teacher created an environment of participation *

27. The teacher maintained a cordial relationship with the entire group of students *

28. The teacher created a climate of trust and productivity in class *

29. The teacher managed to increase interest in the subject

30. The teacher was approachable and had an attitude of availability outside of class *

31. The teacher had been given suggestions that he accepted openly

32. The teacher has been concerned about the evolution of students in their careers

* = Items included in the short version

(Spanish)

**Planificación, dominio y explicación de la asignatura**

1. El profesor expresó convenientemente los objetivos y temas, indicando su relación con la formación profesional de los estudios cursados*
2. El profesor seleccionó adecuadamente las actividades de clase, en función de los objetivos*
3. El profesor ha sido claro en sus explicaciones y exposiciones
4. El profesor relacionó los conceptos y principios teóricos fundamentales con la práctica*
5. El profesor resuelve bien las dificultades que se presentan
6. El profesor muestra dominio de la materia*
7. El profesor demuestra en el aula que planifica sus clases previamente
8. El profesor es creativo y dinámico en la clase
9. El profesor muestra que está al día en la materia que imparte

**Metodología y Recursos**

1. El profesor preparó material didáctico aparte del libro de texto y lo dio a conocer
2. El profesor organizó experiencias didácticas tales como visitas, excursiones, proyectos, discusiones*
3. Ha sido interesante el material complementario recomendado o utilizado por el profesor*
4. El profesor utiliza medios que favorecen el aprendizaje*
5. El profesor utilizó convenientemente diferentes métodos de enseñanza
6. El profesor ha utilizado una metodología variada*

**Evaluación**

1. El profesor ha explicado los métodos de evaluación del curso
2. El profesor ha utilizado métodos objetivos para evaluar a los alumnos*
3. El profesor ha utilizado la evaluación para reorientar el aprendizaje de los alumnos
4. El profesor en n la evaluación ha tenido en cuenta aspectos que no son meramente cognoscitivos
5. El profesor ha evaluado de forma justa e imparcial*
6. El profesor ha explicado el nivel mínimo para aprobar el curso y porqué*
7. Los objetivos que se pretendían conseguir estaban definidos de modo claro y conciso
8. Los eventos de evaluación guardan relación con la enseñanza impartida*

**Relación profesor-alumno**

1. El profesor comprobó que los alumnos comprendían lo que se les enseñaba
2. El profesor alentó y animó las iniciativas provenientes de los alumnos
3. El profesor creó un ambiente de participación*
4. El profesor mantuvo una relación cordial con todo el grupo de alumnos*
5. El profesor creó un clima de confianza y trabajo en clase*
6. El profesor ha conseguido aumentar el interés por la asignatura
7. El profesor fue asequible, tuvo actitud de disponibilidad fuera de clase*
8. Al profesor se le han hecho sugerencias que aceptó de manera abierta
9. El profesor se ha preocupado por la evolución de los estudiantes en la carrera

* = Items incluidos en la versión corta
